# Hyper‐progressive disease after immune checkpoint inhibitor in SMARCA4‐deficient small‐cell lung carcinoma

**DOI:** 10.1002/rcr2.667

**Published:** 2020-09-21

**Authors:** Yosuke Chiba, Toshinori Kawanami, Kei Yamasaki, Keigo Uchimura, Atsuji Matsuyama, Kazuhiro Yatera

**Affiliations:** ^1^ Department of Respiratory Medicine University of Occupational and Environmental Health Fukuoka Japan; ^2^ Department of Pathology and Oncology University of Occupational and Environmental Health Fukuoka Japan

**Keywords:** Hyper‐progressive disease, lung cancer, nivolumab, SMARCA4‐deficient small‐cell lung carcinoma

## Abstract

SMARCA4 (switch/sucrose non‐fermentable‐related, matrix‐associated, actin‐dependent regulator of chromatin, subfamily A, member 4)‐deficient thoracic tumours have shown poor prognosis in clinical settings. Although the optimal treatment for SMARCA4‐deficient thoracic tumours remains unclear, existing studies indicate a favourable response of these tumours to immune checkpoint inhibitors (ICIs). However, there are no reports of fatality in SMARCA4‐deficient small‐cell lung carcinoma (SCLC) with hyper‐progressive disease (HPD) upon treatment with ICIs. Herein, we report a patient with SMARCA4‐deficient SCLC who had HPD after the first ICI treatment. A 35‐year‐old man was treated with nivolumab, subsequent to cytotoxic chemotherapy. A week after nivolumab initiation, chest computed tomography revealed marked increase in pleural effusion in the right lung and chest wall dissemination of the tumour, which concur with the definition of HPD. This is the first study to report the occurrence of HPD after treatment with ICIs in a patient with SMARCA4‐deficient SCLC. Analysis of additional data is necessary to determine the optimal treatment for these patients.

## Graphical abstract

Herein, we report a patient with SMARCA4 (switch/sucrose non‐fermentable‐related, matrix‐associated, actin‐dependent regulator of chromatin, subfamily A, member 4)‐deficient small‐cell lung carcinoma (SCLC) who had hyper‐progressive disease (HPD) after the first immune checkpoint inhibitors (ICI) treatment. A 35‐year‐old man was treated with nivolumab, subsequent to cytotoxic chemotherapy. A week after nivolumab initiation, chest computed tomography revealed marked increase in pleural effusion in the right lung and chest wall dissemination of the tumour, which concur with the definition of HPD. This is the first study to report the occurrence of HPD after treatment with ICIs in a patient with SMARCA4‐deficient SCLC. Analysis of additional data is necessary to determine the optimal treatment for these patients.

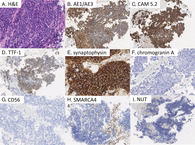

## Introduction

SMARCA4 (switch/sucrose non‐fermentable‐related, matrix‐associated, actin‐dependent regulator of chromatin, subfamily A, member 4) is a catalytic subunit essential for the function of the switch/sucrose non‐fermentable complex that plays important roles in transcription, differentiation, and DNA repair. SMARCA4 mutations were detected in 5% of non‐small cell lung carcinoma (NSCLC) patients [1].

Recently, SMARCA4‐deficient thoracic tumours have clinically been recognized because of its poor prognosis in clinical settings. The correlation of the loss of SMARCA4 with poor prognosis is remarkable in thoracic tumours compared with that in other tumours [1]. Although the optimal treatment for SMARCA4‐deficient thoracic tumours remains unclear, several reports have indicated a beneficial effect of immune checkpoint inhibitors (ICIs) [2]. However, there are no studies that described a fatal case of SMARCA4‐deficient thoracic tumour with hyper‐progressive disease (HPD) manifestations, upon treatment with ICIs. In this novel case study, we report a patient with SMARCA4‐deficient small‐cell lung carcinoma (SCLC) who died due to drastic HPD, consequent upon the first ICI treatment.

## Case Report

A 35‐year‐old male current smoker (22.5 pack‐years) visited our hospital with facial oedema and persistent productive cough. Upon admission to our hospital, physical examination revealed a height of 170 cm, body weight of 51 kg, body temperature of 37.0°C, heart rate of 108 bpm, blood pressure of 107/64 mmHg, and oxygen saturation of 99% (room air, rest). Analysis of chest auscultation revealed attenuated respiratory sounds in the upper right lung field. The laboratory findings were as follows: white blood cells, 9000/mm^3^ with 63.9% neutrophils and 24.2% lymphocytes; haemoglobin, 9.2 g/dL; albumin, 3.4 g/dL; lactate dehydrogenase (LDH), 341 IU/L; calcium, 9.4 mg/dL; C‐reactive protein, 3.32 mg/dL; carcinoembryonic antigen, 122.7 ng/mL; and pro‐gastrin‐releasing peptide, 2850 pg/mL. Chest radiography and high‐resolution computed tomography (HRCT) showed a right hilar lung mass in the right upper lobe with pleural dissemination and multiple hilar, para‐tracheal and subcarinal lymphadenopathies. Fluorodeoxyglucose‐positron emission tomography with CT showed positive accumulation (maximum standardized uptake value = 14.3) in these thoracic lesions with high‐uptake lesions in multiple bones (skull, sacrum, and right acetabulum). The target lesions were decided by Response Evaluation Criteria in Solid Tumours version 1.1 (RECISTv1.1).

**Figure 1:**
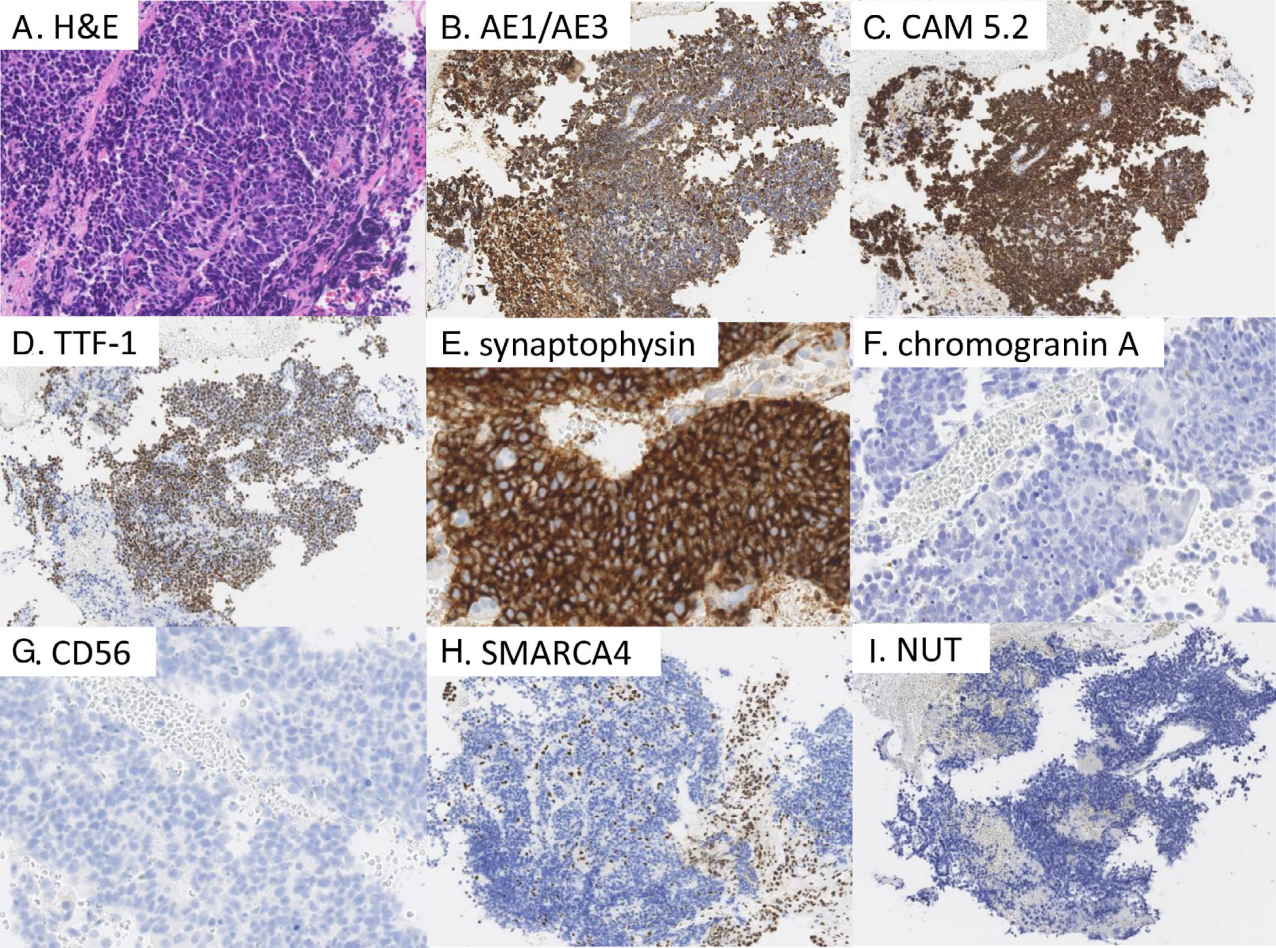
Histopathological findings of the transbronchial biopsy. (A) H&E staining. Undifferentiated carcinoma cells are proliferating in sheets or cords. (B, C) the tumour cells are diffusely positive for cytokeratin (AE1/AE3 and CAM 5.2). (D, E) the tumour cells are diffusely positive for thyroid transcription factor‐1 and synaptophysin. (F, G) The tumour cells are negative for chromogranin A and CD56. (H) Diffusely diminished staining of SMARCA4 is seen in tumour cells. (I) The tumour cells are negative for nuclear protein in the testis (NUT). The overall feature is suggestive of small‐cell lung carcinoma despite some unusual findings such as prominent nucleoli and larger nuclei. Original magnification: (A) ×20, (B–D, H, I) ×10, (E–G) ×40.

Pathological examinations of the right S3 lung tumour under bronchoscopic guidance revealed proliferation of small round cells focally with prominent nucleoli and larger nuclei in sheets, nests, or cords with necrotic foci. The observations such as prominent nucleoli and larger nuclei were unusual, as these were not included in the descriptions of any categories of the World Health Organization classification of NSCLC based on histological types. Thus, the observations from the present case were indicative of SCLC. Immunohistochemistry (Bond MAX Immunostainer, Leica Microsystems) results showed that the tumour cells were diffusely positive for thyroid transcription factor‐1 (Dako), cytokeratin markers (AE1/AE3; Dako, and CAM5.2; Becton Dickinson), and synaptophysin (Dako) and negative for chromogranin A (Dako), CD56 (Leica Biosystems), nuclear protein in the testis (Cell Signalling Technology), and SMARCA4 (Epitomics) (Fig. 1). The tumour proportion score of programmed death‐ligand‐1 (PD‐L1) was 1%–24% according to the 22C3 IHC pharmDX assay (Dako). Molecular analyses revealed no mutations or rearrangements of *EGFR*, *ALK*, or *ROS1*.

The patient was initially treated with two cycles of combined chemotherapy with cisplatin and etoposide, but the thoracic lesions aggravated. The chemotherapy regimens for SCLC were considered ineffective; hence, NSCLC regimens were employed after second‐line treatment considering the patient's atypical pathological and clinical characteristics of SCLC. Carboplatin along with paclitaxel (protein‐bound) was used as the second‐line therapeutic regimen for. After two cycles of chemotherapy with carboplatin plus paclitaxel (protein‐bound) and palliative radiation therapy (right upper lobe: 30 Gy/10 fr and right ilium: 30 Gy/10 fr), the sites of radiotherapy showed response. However, multiple new bone metastases were evident.

**Figure 2:**
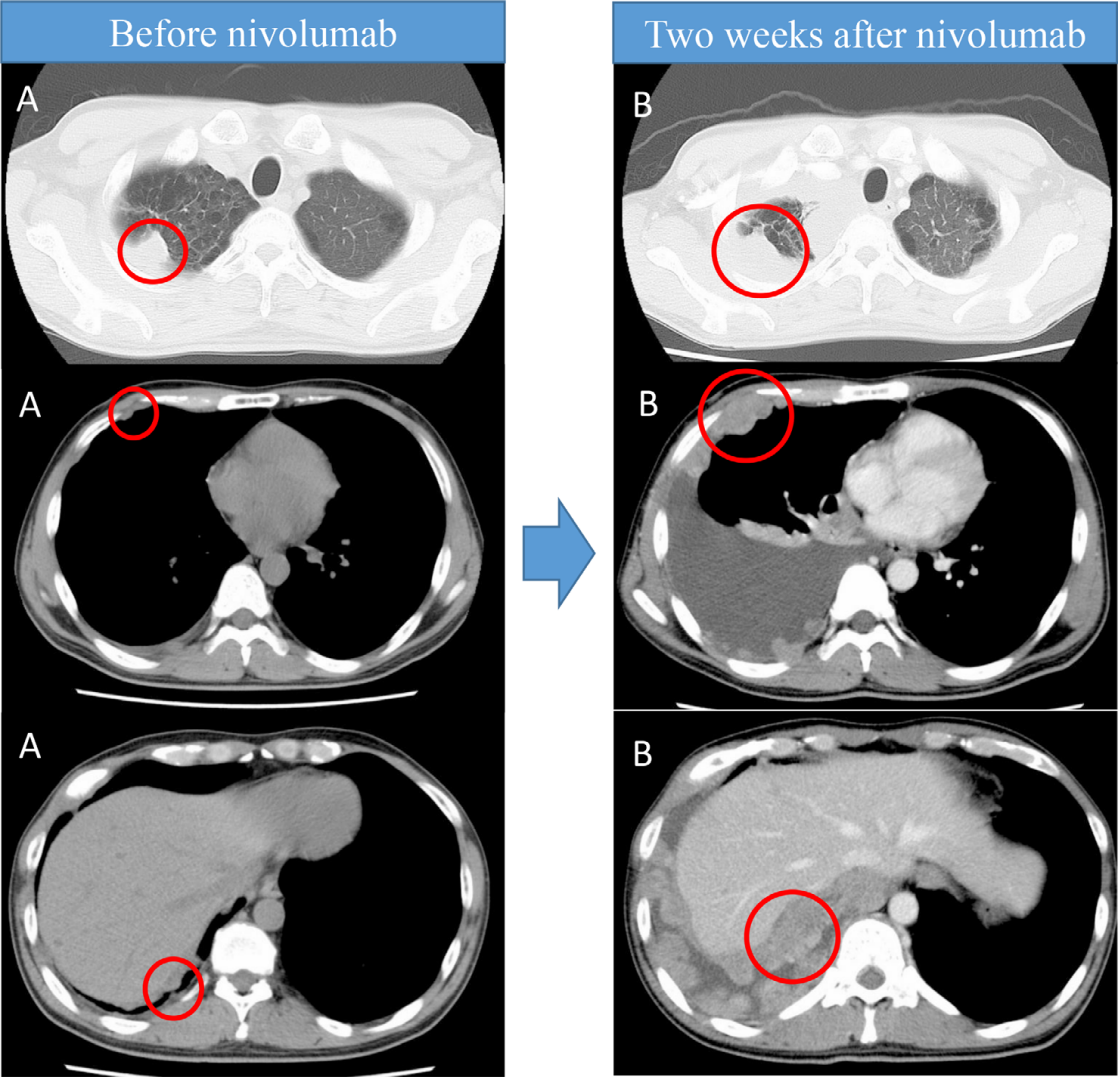
Chest computed tomography (CT) images before nivolumab treatment (A) and two weeks after nivolumab treatment (B), showing increased rapid pleural effusion and dissemination.

The subject was treated with nivolumab as a next‐line treatment. A week after initiating nivolumab, the patient appeared febrile (38–39°C) with pain in the right chest. Chest HRCT performed on day 15 after nivolumab administration showed a marked increase in pleural effusion in the right lung and chest wall dissemination of the tumour (Fig. 2).

HPD was considered to have been caused by treatment with nivolumab. The patient's Eastern Cooperative Oncology Group performance status score had rapidly deteriorated from 1 to 3, necessitating switching of the treatment regimen to docetaxel and ramucirumab. However, tumour progression was uncontrollable, and the patient died due to respiratory failure, 27 days after docetaxel and ramucirumab administration.

## Discussion

Small cell carcinoma is a highly aggressive cancer that arises within the lung, cervix, ovary, prostate, and gastrointestinal tract. SMARCA4‐deficient small cell carcinoma of the ovary, hypercalcaemic type, which is a rare and aggressive form of ovarian cancer, is described to be oncogenically driven exclusively by inactivating mutations in SMARCA4 [3]. However, there are no previous reports on patients with SMARCA4‐deficient SCLC. The pathological discussion of this patient is described in another literature in Japanese [4].

In this patient, platinum‐doublet chemotherapy was ineffective, and the nivolumab therapy caused HPD. HPD is a novel pattern of progression with an unexpected and fast progression in both tumour volume and rate. According to the RECISTv1.1, HPD is indicated by disease progression with tumour growth rate (TGR) exceeding 50% during PD‐1/PD‐L1 inhibitor therapy, and 13.8% of patients with advanced NSCLC treated with PD‐1/PD‐L1 inhibitors had advanced HPD associated with a high number of metastatic sites and poor survival [5]. In our patient, the TGR before and during treatment and the variation per month was 496%, thereby fulfilling the criteria for HPD.

While the aetiology and pathogenesis of HPD are unclear, associations with a lower frequency of effector/memory subsets (CCR7‐CD45RA‐T cells among the total CD8+ T cells) and a higher frequency of severely exhausted populations (TIGIT+ T cells among PD‐1+ CD8+ T cells) have been reported [6]. In a meta‐analysis of HPD during ICI therapy, a high serum level of LDH, having more than two metastatic sites, having liver metastases, a Royal Marsden Hospital (RMH) prognostic score ≥2 (including three factors with a score of 1 each: an elevated LDH level, serum albumin lower than 3.5 g/dL and more than two metastatic sites), and negative PD‐L1 expression were significantly associated with HPD [7]. In the present case, three risk factors (high LDH, more than two metastases, and RMH prognostic score of 2) for HPD were observed.

Although patients with an SMARCA4‐deficient thoracic tumour have an extremely poor survival outcome, there are some reports of patients with an SMARCA4‐deficient thoracic tumour being successfully treated with ICIs. This suggested that ICIs could be effective against SMARCA4‐deficient thoracic tumours [2]. However, the difference between responders and non‐responders to ICIs is unclear. Physicians should therefore carefully consider that the disease may progress rather rapidly upon treatment with ICIs, especially in patients with risk factors that are reportedly associated with HPD.

In conclusion, our patient showed fatal HPD after the first nivolumab. The accumulation of further clinical information is necessary to determine the optimal treatment of SMARCA4‐deficient thoracic tumour.

### Disclosure Statement

Appropriate written informed consent was obtained for publication of this case report and accompanying images.
